# Sensory processing patterns affect headache severity among adolescents with migraine

**DOI:** 10.1186/s10194-020-01119-0

**Published:** 2020-05-06

**Authors:** Jacob Genizi, Ayelet Halevy, Mitchell Schertz, Khaled Osman, Nurit Assaf, Idan Segal, Isaac Srugo, Aharon Kessel, Batya Engel-Yeger

**Affiliations:** 1 Pediatric Neurology Unit, Bnai Zion Medical Center, Haifa, Israel; 2 Department of Pediatrics, Bnai Zion Medical Center, Haifa, Israel; 3 Bruce and Ruth Rappaport Faculty of Medicine, Technion, Haifa, Israel; 4 Department of Pediatric Neurology, Schneider Children’s Medical Center, Petach Tikva, Sackler Faculty of Medicine, Tel Aviv University, Tel Aviv, Israel; 5 Child Development and Pediatric Neurology Service, Meuhedet-Northern Region, Haifa, Israel; 6 Division of Allergy and Clinical Immunology, Bnai Zion Medical Center, Haifa, Israel; 7 Occupational Therapy Department, University of Haifa, Haifa, Israel

**Keywords:** Sensory processing, Pain catastrophizing level, Adolescents, Headache, Migraine

## Abstract

**Objective:**

To evaluate the relationship between pain catastrophizing level, sensory processing patterns, and headache severity among adolescents with episodic migraine.

**Background:**

Catastrophizing about pain is a critical variable in how we understand adjustment to pain and has a unique contribution in predicting pain intensity. Recent reports found that migraine is also related to enhanced sensory sensitivity. However, the relationship between pain severity, pain catastrophizing level and sensory sensitivity requires greater study especially among adolescents.

**Methods:**

Participants were 92 adolescents aged 13–18 years, 40 with episodic migraine and 52 healthy controls. The migraine patients were prospectively recruited from outpatient pediatric neurology clinics. All participants completed the Adolescent/Adult Sensory Profile (AASP), and the Pain Catastrophizing Scale for children (PCS-ch). The migraine groups also completed the PedMIDAS, which measures Headache related disability.

**Results:**

Adolescents with migraine had significantly lower tendency to seek sensory input than healthy controls. Elevated rumination and helplessness correlated with higher migraine pain severity. Tendency to avoid sensory input predicted the migraine related disability level. They also significantly higher pain catastrophizing level than healthy controls, as seen in enhanced rumination (*p* ≤ 0.001) and helplessness (*p* ≤ 0.05).

**Conclusions:**

Sensory processing difficulties are common among adolescents with episodic migraine. Sensory avoidance may be related to pain experience, and pain catastrophizing and disability level.

**Trial registration:**

ISRCTN ISRCTN73824458. Registered 28 September 2014. retrospectively registered.

## Introduction

Headache is a common complaint among children [1], and its prevalence rises dramaticaly during adolescence [2–4]. Migraine headaches may reduce quality of life [5] and cause disability in everyday activities [6]. This is significantly connected to the experience of pain which limits the ability to function normally [7]. The tendency to “catastrophize” during painful experiences contributes to more intense pain and increased emotional stress [8–10]. Recent studies found connection between migraine patients’ function and pain catastrophizing both in adults [11] and children [12]. The level of pain catastrophizing is associated with increased pain experience and pain-related outcomes caused by augmented nociception processing through affective and cognitive processes [13]. The enhanced sensory perception may be related to a general trend of sensory hypersensitivity resulting from sensory processing difficulties (SPD) [14, 15]. Individuals who are more sensitive to sensory information than others often perceive sensory events as noxious and stressful [16].

Migraine in particular, is associated with increased hypersensitivity to various sensory stimuli: visual, auditory, odor and somatosensory, both before aura and during the headache attack [17]. In a previous study [18] we have found that children ages 6–12 years with migraine show extremely different sensory patters as compared to healthy controls, with a strong connection between the altered sensory profile and low quality of life. However, adolescence is a transition period and thus pain during adolescence is an important predictor of future pain [19]. The knowledge about both pain perception and its relation to sensory processing patterns is scarce.

The aims of the present study were: 1: to compare pain catastrophizing level and sensory processing patterns as expressed in daily life between adolescents with migraine and healthy controls. 2: Examine the correlations between sensory processing and pain catastrophizing level in each group as well as the correlations between pain catastrophizing level, migraine related disability among adolescents with migraine. 3: Predict migraine related disability by pain catastrophizing level and sensory processing patterns among adolescents with migraine.

In our study it was hypothesized that (1) adolescents with migraine would have higher prevalence of extreme sensory processing patterns, mainly expressed by hypersensitivity, and higher pain catastrophizing level as compared to healthy controls; (2) in both groups extreme sensory processing patterns would be correlated with enhanced pain catastrophizing level; among adolescents with migraine the sensory processing and pain catastrophizing level would correlate with enhanced migraine pain and disability; (3) Extreme sensory processing and pain catastrophizing level would significantly predict migraine related disability.

## Materials and methods

### Participants

The study included 92 adolescents aged 13–18 years, coming from average and above average socio-economic levels. The study group included 40 participants (19 boys and 21 girls) with episodic migraine (8 patients [20%], had migraine with aura) who were prospectively recruited from the following outpatient pediatric neurology clinics: 1.The pediatric neurology clinics at the Bnai- Zion Medical Center, 2. The pediatric neurology clinics at the Schneider Children’s Medical Center, Petach Tikva and 3. The pediatric neurology clinics at the Meuhedet Medical Services in the city of Haifa, during the years 2014–2017. The control group included 52 healthy children, 21 boys and 31 girls, who did not have any significant illness or migraine; did not have positive neurological findings or developmental disorders. Table 1 summarizes the study and control groups’ demographic information. (Table 1).

**Table 1:** Participants’ health and demographic information

		Adolescents with Migraine (*n* = 40)	Healthy Controls (*n* = 52)
Mean age **±** SD		15.03 ± 1.45	15.27 **±** 1.62
Gender - n (%)	Boys	19 (47.5%)	21 (40.4%)
	Girls	21 (52.5%)	31 (59.6%)
MIDAS level (%)	No functional impairments	22.5%	
	Minimal functional impairments	20%	
	Moderate functional impairments	15%	
	Severe functional impairments	27.5%	
	missing	15%	
VAS (range, mean ± SD)	0–10, 7.81 ± 2.27		

### Methods

#### Medical assessment

A prospective medical history including a thorough headache history and physical and neurological assessment by a pediatric neurologist, were done in all children, during the visit at the pediatric neurology clinic. All children met the diagnostic criteria for migraine, according to the International Classification of Headache Disorders, 3rd edition (ICHD-3 beta) [20]. Allodynia was not formally assessed.

#### PedMIDAS

Headache related disability was evaluated by the PedMIDAS questionnaire. It was developed to assess migraine disability in pediatric and adolescent patients and has been tested and validated for ages 4 to 18 [21].

*Pain catastrophizing scale for children (PCS-C)* [22] - assesses catastrophizing about pain in children and their parents. In addition to the total score, three scores are generated: rumination, magnification and feelings of helplessness. This questionnaire has good psychometric properties [22].

*The Adolescent/Adult Sensory Profile (AASP)* [23] – a self-report that measures behavioural responses to sensory input in daily life. The AASP includes 60 items, sorted into the four quadrants of Dunns’ model of sensory processing [24]: Low Registration (for example: don’t seem to notice when face of hands are dirty), Sensation Seeking (for example: touch others while talking to them; hum, whistle, sing or make other noises) Sensory Sensitivity (for example: startle easily at unexpected or load noises) and Sensation Avoidance (for example: stay away from crowds; stay away from noisy settings). Participants indicate how often they respond to the sensory event on a 5-point Likert scale (1 = “almost never” to 5 = “almost always”). The resultant score for each quadrant ranges from 5 to 75. Norms were defined for various age groups (11–17; 18–64; 65 and older).

### Procedure

The study group patients were recruited during their visit at the neurology clinics as described above. All patients agreed to participate in the study and their parents signed an informed consent. The headache history was taken and the neurological examination was performed during the visit. The diagnosis of migraine was made according to ICHD-3 beta [20]. Patients completed the PedMIDAS questionnaire, the PCS-C and the AASP.

Adolescents from the control group were recruited after their parents answered an advertisements calling to participate in the study by contacting the study conductor, and after approving their answer. The controls were evaluated in their homes and also completed the PCS-C and the AASP after they and their parents signed consent forms.

The study received ethical approval from the Bnai Zion Medical Center Ethics Review Board 21–14-BNZ.

### Data analysis

Normality tests were applied and most dependent variables showed normal distribution. For examining hypothesis 1: Chi square analysis examined whether significant differences exist between groups in the percentage of children found in each of the AASP quadrant (less than most people = less than normal; similar to most people = normal; more than most people = above than normal. Differences between groups in subscale scores of PCS-C and AASP were examined by Multivariate analysis of variance (MANOVA). Difference in PCS-C total score between groups was examined by independent t-test. The dependent variables were the AASP and PCS-C scores. For examining hypothesis 2, Pearson test examined the correlations between the variables. The dependent variables were the PCS-C scores. For examining hypothesis 3, a multiple linear regression with stepwise method was performed to evaluate the ability of PCS-C and AASP scores to predict migraine disability level (measured by PedMIDAS). The independent variables were: PCS-C total score which was entered to the first step, and the four sensory patterns of the AASP which were included in the second step. The dependent variable was PedMIDAS. The level of significance was set at *p* ≤ .05.

## Results

### Comparing pain catastrophizing level between adolescents with migraine and healthy controls

Adolescents with migraine had significantly higher rumination (*p* ≤ 0.001) and helplessness (*p* ≤ 0.05) than healthy controls. The difference between both groups was also expressed in the total PCS-C score. Although Magnification was higher among adolescents with migraine, the difference between groups was not significant (Table 2).

**Table 2:** Comparing PCS- C scores between adolescents with Migraine and healthy controls

	Adolescents with Migraine (*n* = 40)			Healthy Controls (*n* = 52)			
	Mean	SD	Range	Mean	SD	Range	
Rumination	7.05	3.18	0–12	4.82	2.67	0–10	F _1,88=_12.01***
Magnification	4.47	2.91	0–12	4.03	2.43	0–9	F _1,88=_.836
Helplessness	10.89	6.12	0–23	8.55	4.33	0–17	F _1,88=_4.51*
Total	22.55	10.87	0–45	17.42	7.69	0–31	t_90_ = −2.45**

*p* ≤ 0.05*; *p* ≤ 0.01**; *p* ≤ 0.001***

“F” represents the Multivariate analysis of variance

“t” represents the intendent t-test results

### Comparing the sensory processing patterns between adolescents with migraine and healthy controls

Adolescents with migraine had significantly lower tendency to seek sensory input than the healthy controls. 31% of the study group were in the under norm in the seeking quadrant as compared to 17% of the health controls (Chi square = 5.87, *p* = .05). In all other sensory profiles, no significant differences were found between both groups (Table 3). However, the prevalence of sensory sensitivity and avoidance was higher among the study group as compared to the healthy controls: 11.5% of the study group were above norm range in sensory sensitivity as compared to 7.7% of the controls; 31.4% of the study group were above norm range in Sensation avoidance as compared to 23.1% of the controls.

**Table 3:** Comparing the sensory processing patterns (according to the AASP) between adolescents with Migraine and healthy controls

	Adolescents with Migraine (*n* = 38)			Healthy Controls (*n* = 52)			
AASP profiles	Mean	SD	Range	Mean	SD	Range	F _1,85_
Low Registration	28.91	8.91	15–53	27.62	4.94	18–40	.76
Sensory Seeking	42.42	7.34	24–53	48.56	7.19	32–62	14.93***
Sensory Sensitivity	36.88	10.18	18–63	35.13	6.32	20–53	.98
Sensation Avoiding	31.62	7.63	16–53	30.69	5.99	21–48	.38

*p* ≤ 0.001***

higher scores indicate worse sensory processing

### The correlations between sensory processing patterns and pain catastrophizing scores in both groups

Among the healthy controls, greater sensory sensitivity and avoidance correlated with elevated rumination (*r* = .38, *p* = .006; *r* = .37, *p* = .006, respectively) and with elevated helplessness (*r* = .31, *p* = .03; *r* = .40, *p* = .004, respectively). Among the study group, only one significant correlation was found – elevated magnification correlated with greater sensory avoidance (*r* = .39, *p* = .02).

### The correlations between pain catastrophizing, migraine pain severity and related disability (MIDAS score) among adolescents with migraine

Elevated rumination and helplessness correlated with higher migraine pain severity (r = .48, *p* = .03; *r* = .49, *p* = .02, respectively).

### Predicting the migraine related disability (MIDAS score) in adolescents with migraine by their total PCS-C score and the sensory profiles

The only significant predictor of migraine related disability was sensory avoidance – greater tendency to avoid sensory input accounted for 26% of the variance (F_1,28=_10.019; B = 3.36; SE B = 1.01; β = .51, *p* = 0.04).

## Discussion

The main finding of the present study is that adolescents with episodic migraine had significantly lower tendency to seek sensory input compared to healthy controls.

Although both groups did not significantly differ in sensory sensitivity and avoidance, the prevalence of adolescents with migraine found above norm values in both of these sensory patterns was twice higher. We have demonstrated that sensory avoidance was a predictor of migraine related disability, as reflected in the PedMIDAS score.

The possible connection between migraine and sensory processing patterns as expressed in daily living scenarios among patients with migraine has only rarely been reported. Nahman-Averbuch et al. [25] in a meta-analysis study, revealed a lower heat and pressure pain thresholds and higher pain ratings to cold stimuli, among patients with migraine. The perception of sensory stimuli such as sound, light, odors and somatosensory stimuli tend to be enhanced among patients with migraine [16, 26–28] between migraine attacks and might even be a trigger to migraine attacks. Coppola et al., [29] found abnormal cortical responses to light and Sand et al. [30] demonstrated, that different high frequency oscillations of the somatosensory evoked potential among migraine patients compared to controls. Noseda [31] found habituation difficulties among patients with migraine, as well as enhanced sensory sensitivity, using Quantitative Sensory Testing (QST) noting that patients with migraine may have greater reactivity to pain.

Studies about SPD in children also found habitation difficulties as measured in the Short Sensory Profile questionnaire [32] and in electrophysiological measures such as the electrodermal reactivity (EDR) [33]. Similar results were found in adults with SPD as manifested by the AASP and evoked response potentials [34].

These findings support the hypothesis that patients with migraine have abnormalities in sensory processing and integration [35]. Recently, Goadsby [36] found that both the aura and the migraine attack, may represent a form of hypersensitivity due to sensory processing difficulties. Mainero et al. [37] demonstrated that patients with migraine have stronger connectivity between the ventrolateral periaqueductal gray (PAG) and other brain areas that are involved in nociceptive and somatosensory processing. Other authors [38] suggested that sensory hypersensitivity may result from activation of subcortical brain areas receiving convergent inputs and then project to different cortical brain areas involved in integrating multiple sensory modalities such as visual, auditory and olfactory. As suggested by Tommaso et al., [35] studies should further explore sensory processing in patients with migraine. These studies should implement objective measures such as neuroimaging to reflect temporal patterns of sensory processing in patients with migraine and correlated them with the accompanying anatomical and functional changes.

Yet, these findings are related to adults with migraine, and less is known about extreme sensory processing among younger patients with migraine. In a previous study [18] we also found that young children (6–12 years of age) with migraine had greater prevalence of extreme sensory processing patterns, expressed in hypersensitivity, which also correlated with their low quality of life. The negative effects that extreme sensory processing patterns may have on daily function [39] and quality of life [18], and their prevalence among children, emphasize the need to elaborate the knowledge about this relationship in adolescents as well and explore the association between sensory processing patterns, pain experience – catastrophization and related disability.

In the present study, the pain catastrophizing scale for children (PCS-C) was used to measure the functional and psychological consequences of pain among adolescents with migraine. Catastrophizing about pain is a critical variable in how we understand adjustment to pain and has a unique contribution in predicting pain intensity. The theoretical bases were defined by Sullivan et al. [9] who considered catastrophizing as a part of the appraisal model [40] that described rumination and magnification as primary appraisal processes in which patients place emphasis on the fear from pain sensations. Helplessness is related to secondary appraisal processes in which patients under evaluate their ability to manage pain effectively. Magnification and rumination usually cause pain avoidance.

In the present study, among adolescents with migraine, pain magnification correlated with sensory avoidance, probably because both factors represent the same hypersensitivity - to non-aversive stimuli of daily scenarios as well as to painful stimuli. A main characterizes of individuals with sensory hypersensitivity is their magnification of the sensation, the inability to control the overwhelming sensation and adapt to it similar to people with pain catastrophizing [8]. In line with the “Appraisal Model” [41] the ineffective coping with threatful sensory stimuli and the inability to use effective coping strategy to manage and adapt to this inconvenience, characterizes both individuals with pain catastrophizing, with sensory hypersensitivity [42], and thus in individuals with migraine.

Recently, Sciruicchio et al. [12] evaluated pain catastrophizing among children with migraine and found no difference in total pain catastrophizing score (PCS-C) between children with episodic versus chronic migraine. In our study, adolescents with episodic migraine had significantly higher rates of rumination and helplessness and this may affect their pain experience, fear and lead to lower tendency to seek for sensory input. Sciruicchio et al. [12] also reported that PCS-C did not correlate with the PedMIDAS score. In our study migraine severity correlated with elevated rumination. It might be effective to refer to the PCS-C scales and not only to the total scores, in order to better understand what are the pain catastrophizing parameters that play a role in migraine. How they are related to other characteristics of individuals with migraine, such as their sensory processing patterns, and how it is related to their daily function.

These findings, together with our current report on the connection between migraine and sensory avoidance, raises a new prospective to migraine treatment in adolescents. Intervention programs should consider anxiety or depressive disorders and other forms of psychopathology in adolescence with migraine [43–45] with respect to extreme sensory processing patterns. The extreme sensory processing patterns may be related to the low academic performance and school refusal, as well as with somatic and emotional complaints in adolescents with migraine [46]. Intervention, focusing on coping strategies to deal with pain perception and the extreme sensory processing patterns as expressed in daily scenarios should be applied to optimize function and quality of life. By that, the negative consequences of migraine and related difficulties in terms of social, academic and personal adjustment may be reduced [47, 48].

### Limitations

The study consisted on a relatively small sample. Although patterns of sensory sensitivity and avoidance were not significantly different between both groups, the larger children with migraine found above norms in these patterns, raise the need to further examine the relationships between these sensory patterns and pain perception on larger samples in order to enable generalizability of the results.

## Conclusions

Enhanced pain catastrophizing level and extreme sensory processing patterns appear to characterize adolescents with episodic migraine. Lower tendency to seek sensory input may predict migraine related disability. Based on the negative functional and psychological effects of episodic migraine in adolescents, research and practice in migraine should incorporate pain perception and sensory processing, especially as it relates to daily function and quality of life.

## Data Availability

All data and material are available.
